# Prenatal sugar exposure shapes late-life human capital and health

**DOI:** 10.1093/pnasnexus/pgaf301

**Published:** 2025-09-20

**Authors:** Gerard J van den Berg, Stephanie von Hinke, R Adele H Wang

**Affiliations:** Faculty of Economics and Business, University of Groningen, 9747 AD Groningen, The Netherlands; UMC Groningen, 9713 GZ Groningen, The Netherlands; IFAU Uppsala, 75312 Uppsala, Sweden; ZEW, 68161 Mannheim, Germany; J-PAL, Cambridge, MA 02142, USA; CEPR, London EX1R 5HL, United Kingdom; School of Economics, University of Bristol, Bristol BS8 1TU, United Kingdom; IFS, London NW1 4DF, United Kingdom; IZA, 53113 Bonn, Germany; School of Economics, University of Bristol, Bristol BS8 1TU, United Kingdom

**Keywords:** nutrition, food consumption, gene–environment interplay, developmental origins

## Abstract

Maternal sugar consumption during pregnancy may have a variety of effects on offspring. We exploit the abolishment of the rationing of sweet confectionery in the United Kingdom on 1949 April 24, and its subsequent reintroduction some months later, in an era of otherwise uninterrupted rationing of confectionery (1942–1953), sugar (1940–1953), and many other foods, and we consider effects on late-life cardiovascular disease, body mass index (BMI), height, type-2 diabetes, and the intake of sugar, fat, and carbohydrates, as well as cognitive outcomes and birth weight. We use individual-level data from the UK Biobank for cohorts born between April 1947 and May 1952. We also explore whether one's genetic “predisposition” to the outcome can moderate the effects of prenatal sugar exposure. We find that prenatal exposure to derationing increases education and reduces BMI and sugar consumption at higher ages, in line with the “developmental origins” explanatory framework, and that the sugar effects are stronger for those who are genetically “predisposed” to sugar consumption.

Significance Statement We show that prenatal exposure to the temporary abolition of confectionery rationing in 1949 led to increases in education and reductions in body mass index and sugar consumption at higher ages. In line with the idea of fetal programming, the sugar effects are stronger for those who are genetically “predisposed” to sugar consumption.

## Introduction

Sugar has been part of the human diet for centuries, providing energy and making food more palatable. More recently, sugar consumption has been related to a range of diet-related health problems. The WHO recommends that individuals’ intake of sugar is restricted to <10% of total energy intake (1), justified by countless studies of adults and children, documenting adverse (health) effects due to short-to-medium-term sugar exposure. Much less is known about the effects of exposure to sugars in utero and in particular, about long-term effects on health at advanced ages or on human capital outcomes. Following the literature on the developmental origins of late-life health, one may hypothesize the existence of in utero exposure effects. At first sight, this literature suggests that a high intake helps to prevent undernutrition and provides an in utero environment that is better aligned with later-life nutritional conditions, improving late-life health (2–7). However, this ignores the specific nature of sugar as a nutrient with problematic aspects. Sugar exposure may adversely affect the buildup of the body, leading to worse late-life health. This second pathway is supported by the fact that the set of late-life health outcomes that are known to be responsive to adverse in utero conditions in general (such as cardiovascular disease, type-2 diabetes and cognitive decline) closely resembles the set of health outcomes responsive to abundant consumption of sugars. In either case, knowing whether causal long-run effects exist is important for public policy.

A number of descriptive studies have examined the association between maternal gestational sugar consumption and offspring early-life health outcomes. A higher intake of sugars (e.g. as sweets, snacks and soft drinks) tends to be associated with less healthy outcomes, although the evidence is not unanimous (8–13). These studies, however, do not consider late-life outcomes, and, crucially, they do not account for the fact that sugar consumption is a choice and as a result, will correlate with a wide range of parent\al characteristics and circumstances. Many of those characteristics also affect other choices and behaviors, thus influencing the outcomes of their offspring. Any association between sugar intake and offspring health may therefore reflect shared unobserved determinants.

This paper takes a different approach to study the effects of in utero exposure to sugar on late-life health and human capital outcomes using the UK Biobank (UKB) (14). We use a natural experiment, exploiting features of the food rationing system in the United Kingdom in the years immediately following World War II: we exploit the abolition of the rationing of confectionery (i.e. chocolate and sweets) in the United Kingdom on 1949 April 24 and its subsequent reintroduction on 1949 August 13. In a nutshell, we compare UKB participants who were in utero during the short derationing period with those who were in utero just before or after. Our quasi-experimental design, comparing individuals born just a few months apart but exposed to different prenatal conditions, therefore strengthens a likely causal interpretation of our findings. Note that our approach does not discard the importance of other prenatal or postnatal factors that shape individuals’ health throughout life. Instead, the validity of our design relies on the lack of correlation between these factors and the variation exploited by the temporary confectionery derationing.

Our setting has four main advantages. First, the “derationing period” occurred in an era of otherwise uninterrupted rationing of confectionery (1942–1953) and of raw sugar (1940–1953) as well as many other foods, and it happened in a period without other major rationing abolitions/introductions. The widespread rationing of other foods means that confectionery derationing is unlikely to have caused changes in the consumption of other foods/nutrients, simply because these faced an ongoing binding constraint. Hence, its introduction was exogenous, precisely delineated in time, and difficult to anticipate at the individual level (see (15) and Appendix A in the Supplementary Material for detail about UK rationing and about confectionery rationing in particular).

Second, the exposure period is substantially shorter than the length of gestation, allowing us to identify gestational ages that are particularly sensitive to high exposure, akin to the Dutch Hunger Winter studies (5, 16). Study designs based on a single discontinuity do not have this advantage. Third, the maternal dietary changes induced by the derationing are relatively mild compared with major shocks (such as famines) that are commonly exploited in the causal inference literature on conditions in utero. This makes our analysis less sensitive to selective fertility issues that have plagued the latter literature. Fourth, sugar and confectionery consumption is amenable to specific intervenable policy targets, whereas nutritional policy motivated by famine studies requires extrapolations from extreme events.

As motivated by the literature, our set of late-life outcome variables includes educational attainment, cardiovascular disease, body mass index (BMI), height, type-2 diabetes, and the intake of sugar, fat, and carbohydrates. We also examine effects on birth weight. We investigate the direct effect of prenatal exposure to sugar derationing on individual outcomes, but we also explore whether one's genetic “predisposition” can moderate the effects of prenatal exposure. We use individuals’ polygenic indices for the respective health and education outcomes as measures of the genetic “predisposition” for these traits (see Appendix B for more information on their construction). Historically, the study of gene-by-environment (*G* × *E*) interactions is hampered by possible confounders of the environment due to gene–environment correlation (*rGE*) (17). Natural experiments like ours can provide a solution (see also, e.g. (18–25)).

## Results

The data are presented in Appendix C, showing descriptive statistics of our main outcomes of interest (Table S2), their trends by year-month of birth (Fig. S2), and the absence of gene–environment correlation (*rGE*) for the outcomes and their respective polygenic indices (Fig. S3).

Table 1 presents the baseline results. Panel A presents the estimates from regressions that exclude area fixed effects, while panels B, C, and D report the results from regressions that include county (of which there are 65), administrative county (232), and district of birth (1,434) fixed effects, respectively. Panel A suggests that being in utero during derationing (i.e. more maternal confectionery consumption) increases years of education by 0.16; ∼2 months on an annual base. Note that we control for season of birth. We also find that BMI is 0.09 units lower (0.25 kg for an individual of average height) and sugar intake is 1% or 0.2 percentage points less for those exposed compared with those not exposed.

**Table 1.** Effects of prenatal exposure to confectionery derationing.

	(1)	(2)	(3)	(4)	(5)	(6)	(7)	(8)	(9)
	Years of education	Birth weight (g)	BMI	Height (cm)	CVD	Type II diabetes	Sugar intake	Carb intake	Fats intake
Panel A: no area fixed effects									
E: in utero during derationing	0.157*** (0.047)	14.918 (9.608)	−0.095* (0.051)	0.013 (0.066)	−0.003 (0.006)	−0.003 (0.002)	−0.002* (0.001)	−0.002 (0.001)	−0.001 (0.001)
Panel B: county fixed effects (*n* = 65)									
E: in utero during derationing	0.144*** (0.050)	15.599* (8.182)	−0.089** (0.035)	−0.001 (0.049)	−0.002 (0.006)	−0.003 (0.002)	−0.002 (0.001)	−0.001 (0.001)	−0.001 (0.001)
Panel C: admin. county fixed effects (*n* = 232)									
E: in utero during derationing	0.138*** (0.050)	15.422 (10.238)	−0.083** (0.042)	−0.005 (0.066)	−0.002 (0.006)	−0.003 (0.002)	−0.002** (0.001)	−0.002 (0.001)	−0.001 (0.001)
Panel D: district fixed effects (n = 1434)									
E: in utero during derationing	0.137*** (0.048)	14.708 (9.659)	−0.087** (0.042)	0.000 (0.064)	−0.002 (0.006)	−0.003 (0.002)	−0.002** (0.001)	−0.002 (0.001)	−0.001 (0.001)
Mean of outcome	14.82	3331.9	27.58	168.51	0.51	0.05	0.23	0.48	0.33
No. of observations	84,165	47,452	84,513	84,600	84,631	84,596	36,127	36,127	36,127

Estimates from Eq. (1), with the treatment dummy (*E*) specified as those born between 1949 April 24 and 1950 May 12. CVD, cardiovascular disease. Robust standard errors (clustered by year-month in Panel A and by geographic area in the Panels B–D) in parentheses. *P  *<* 0.10, **P  *<* 0.05, ***P  *<* 0.01.

We find no statistically significant effects on height, cardiovascular disease, and type-2 diabetes or on carbohydrate or fat intake. Indeed, most of these coefficients are relatively precisely estimated at values close to zero. With regard to birth weight, we find a 15 g increase for those exposed to confectionery derationing, although this is not statistically significant in Panel A. Controlling for county (Panel B), administrative county (Panel C), or district of birth (Panel D) fixed effects does not affect our estimates in a notable way.

We next explore whether those who are genetically “predisposed” to a trait are differentially affected by the prenatal exposure. Panels A and B of Table 2 present the estimates without and with administrative county fixed effects, respectively. The additive effects of exposure to derationing are very similar to those in Table 1. The additive effects of the respective polygenic indices are strongly positive for all outcomes. A 1 SD increase in the polygenic index for education and birth weight, for example, is associated with an increase of 1.2–1.3 years of schooling and ∼100 g, respectively.

**Table 2.** G×E effect of prenatal exposure to confectionery derationing.

	(1)	(2)	(3)	(4)	(5)	(6)	(7)	(8)	(9)
	Years of education	Birth weight (g)	BMI	Height (cm)	CVD	Type II diabetes	Sugar intake	Carb intake	Fats intake
Panel A: without area fixed effects									
E: in utero during derationing	0.155*** (0.047)	15.376* (9.224)	−0.083 (0.052)	0.026 (0.055)	−0.002 (0.006)	−0.003 (0.002)	−0.002* (0.001)	−0.001 (0.001)	−0.001 (0.001)
PGI (UKB)	1.295*** (0.017)	101.815*** (3.445)	1.549*** (0.018)	3.407*** (0.021)	0.056*** (0.002)	0.021*** (0.001)	0.004*** (0.000)	0.005*** (0.000)	0.003*** (0.000)
G×E	−0.10 (0.044)	7.854 (8.172)	−0.020 (0.031)	−0.038 (0.042)	−0.006 (0.004)	−0.000 (0.002)	−0.002** (0.001)	−0.003** (0.001)	0.000 (0.001)
Panel B: admin. county fixed effects									
E: in utero during derationing	0.141*** (0.048)	15.821 (10.237)	−0.075* (0.040)	0.015 (0.055)	−0.002 (0.006)	−0.003 (0.002)	−0.002* (0.001)	−0.002 (0.001)	−0.001 (0.001)
PGI (UKB)	1.196*** (0.020)	101.620*** (3.200)	1.531*** (0.019)	3.370*** (0.017)	0.055*** (0.002)	0.020*** (0.001)	0.004*** (0.000)	0.005*** (0.001)	0.003*** (0.000)
G×E	−0.12 (0.035)	8.470 (7.445)	−0.021 (0.037)	−0.030 (0.049)	−0.006* (0.004)	−0.000 (0.002)	−0.002** (0.001)	−0.003** (0.001)	0.000 (0.001)
Mean of outcome	14.82	3331.9	27.58	168.51	0.51	0.05	0.23	0.48	0.33
No. of observations	84,165	47,452	84,513	84,600	84,631	84,596	36,127	36,127	36,127

Estimates from Eq. 2, with the treatment dummy (E) specified as those born between 1949 April 24 and 1950 May 12 and the polygenic index G obtained from using the summary statistics from our tailor-made GWAS on the excluded UKB sample and any siblings. Panel A reports the analysis without controlling for the area of birth fixed effects; Panel B reports the analysis that controls for administrative county of birth fixed effects. CVD, cardiovascular disease. Robust standard errors (clustered by year-month in Panel A and by administrative county in Panel B) in parentheses. *P < 0.10, **P < 0.05, ***P < 0.01.

More importantly, we find evidence of G×E effects. Consider the sugar density in older age for individuals with an average polygenic index (i.e. of 0). This density is 0.2 percentage points lower among those prenatally exposed to derationing compared with those not exposed. Table 2 shows that this decrease is larger than 0.2 for those with a higher polygenic index for sugar. In other words, while a 1 SD increase in the polygenic index for sugar increases individuals’ intake by 0.4 percentage points, this effect is ∼50% (i.e. 0.2 percentage points) smaller among those exposed to prenatal derationing. For the other outcomes, we find no heterogeneous response of exposure to derationing with respect to one's genetic “predisposition” for the trait. Indeed, the interaction effects for these specifications are close to zero and relatively precisely estimated.

We next explore a series of sensitivity checks to ensure that our analysis is robust to different assumptions; these are presented in Appendix D. We start by showing that the positive effect on years of education is largely driven by third trimester exposure, while the (marginally significant) increase in birth weight is due to exposure in the first and second trimester, and the decrease in BMI is due to exposure in the second and third trimester (Appendix D: Section D.1). We also explore the robustness of our estimates to the timing of the reintroduction of confectionery rationing, since there is evidence that some confectionery shops had to close in May 1949 because they ran out of stock (see Appendix A and Appendix D: Section D.2). Furthermore, we show that a *longer* exposure to derationing caused larger effects (Appendix D: Section D.3). We also explore the robustness with respect to the timing of pregnancy, only including those who were already conceived at the announcement of derationing in February 1949 to avoid selection into pregnancy driven by the change in policy (Appendix D: Section D.4). To ensure that our analyses capture the impact of in utero exposure to confectionery derationing rather than other confounders, we also run three placebo (or negative control) analyses, which we discuss in Appendix D: Section D.6. Finally, we show that our results are robust to alternative functional forms for the calendar-time trends (Appendix D: Section D.5), and we find some heterogeneity by gender (Appendix D: Section D.7).

## Discussion

Because of the high rates of obesity and diet-related health problems in the developed world, the potential consequences of excessive sugar intake have received much attention in academic research. Most of this, however, explores the *short-term* health benefits of reduced sugar intake. The *longer-term* effects on health and well-being are much less known, and there is very little evidence on the long-term impacts of sugar exposure in utero.

Our analysis investigates this by exploiting a temporary increase in the availability of confectionery in a period that was characterized by a mostly time-invariant availability of other foods and where the nutritional composition of diets was constant due to most foods being rationed. Furthermore, after temporary exposure in utero and returning to a low sugar environment afterward, individuals in our sample are re-exposed to high sugar from the year 1953 onward. We find that the prenatal exposure to confectionery derationing increased education by ∼0.15 years (1.8 months), lowered later-life BMI by 0.08 units, and reduced later-life sugar consumption by 0.2 percentage points (0.9%), with some evidence that it also led to an increase in birth weights of ∼10–15 g. These are small effects, but these are not unexpected in the sense that we only examine the impacts of a short-term increased availability of confectionery. Indeed, we would not expect to see major changes in individuals’ later-life outcomes. Our analysis, however, emphasizes that even such small changes can have long-term impacts. Another interesting finding is that, despite a small effect on birth weight, we find stronger impacts on outcomes in later life. This is consistent with the literature on, for example, the Dutch hunger winter—a much larger dietary shock than ours—which has been shown to affect chronic disease in older age without necessarily affecting birth weight (26).

The results on BMI and sugar intake, and to some extent those on education and birth weight, are in line with the “fetal programming” or “developmental origins of health and disease” framework to explain later-life health outcomes. This stipulates that late-life health may benefit from a pre- and neonatal environment that is well aligned to the later-life environment. Along these lines, a high-sugar in utero environment is better aligned with abundant nutrition later in life, thereby “programming” the fetus to better cope with such environments and improving its later-life outcomes.

Essentially the same argument can be made when considering confectionery intake during pregnancy as a means to reduce stress. In the absence of confectionery, cravings during pregnancy may not be met, which may increase maternal stress levels and prepare the fetus for a more stressful life. This could decrease birth weight and increase BMI and sugar intake later in life. Indeed, recent research on mice has shown that sugar intake of adults increases the activation of tissue-resident memory T-cells in the intestinal wall, leading to a faster clearance of infections (27). Among pregnant women, this mechanism may explain cravings for sweet food. Either way, according to the developmental origins framework, a lower disease occurrence during gestation protects offspring against adverse health outcomes later in life. Such a mechanism may amplify this stress-related pathway.

The programming explanation is more difficult to reconcile with the absence of effects on certain other outcome variables, including height, cardiovascular disease, and type-2 diabetes, since these have been found to be most responsive to early-life conditions in the literature. However, in contrast to the literature on long-term effects of early-life conditions, we are not analyzing omnibus measures of adversity (such as famines or recessions). Instead, we focus on access to a highly specific food type. The fact that we find effects on later-life sugar intake suggests that adult food preferences depend on the in utero environment. This may, in turn, be a mediator of effects on other outcomes. We view it as an important topic for future research to examine long-term effects of a wide range of specific food types in order to enhance our understanding of their roles in affecting later-life outcomes.

An important advantage of our setting is that individuals were unlikely to substitute or complement the increase in confectionery with other foods, since most other foods were rationed. This implies that we identify the effects of increased prenatal exposure to confectionery per se, without the potential simultaneous adjustments to other food items. Having said that, it is possible that our estimates partially capture a reduced exposure to artificial sweeteners. Indeed, during the rationing period, sugar may have been substituted with saccharin as well as barley extract and licorice (28). This is relevant for our study design insofar that such substitutes may affect health at higher ages. The literature has reported evidence of a positive association between the gestational intake of artificial sweeteners and infant and child obesity (as well as an increased preference for sweet tastes), speculating that this is explained by the effects of sweeteners on the gut microbiome of the child (12, 13). It is not clear whether this extends to late-life health, but (29) report long-term effects along these lines in animal studies.

A slightly different explanation for our findings is that access to confectionery boosted pregnant women's happiness, which, in turn, could have led to an improvement in bonding with the newborn and hence more favorable educational and late-life health outcomes of the offspring. Indeed, the consumption of sugar has been associated with reduced stress-induced cortisol (30), and prenatal stress has been linked to an increase in adverse child outcomes (31, 32). Therefore, a higher consumption of sweets could have provided the mother with psychological benefits with subsequent effects on their children. In this case, the nutritional value of confectionery is not of prime relevance.

A different question that remains is whether null effects on health are, in fact, negative impacts that are offset by potential health improvements driven by increased years of education. Or, alternatively, whether improvements in later life health can be explained by the increase in years of schooling. The literature on the causal impacts of education on health is not unambiguous on such potential mechanisms. Some show that education improves important health outcomes, while others find no evidence of major effects (for a recent review, see (33)).

It is worth highlighting that all individuals in our sample have been exposed to permanent derationing in 1953, although at different ages. The literature on long-term effects of early-life conditions generally finds that the effects of in utero exposure exceed those of exposure after birth. We also point out that exposures after 1953 are fully in line with our aim to study effects within the fetal hypothesis framework: estimating the effects of prenatal exposure for those who, later on, live in a society with abundant nutrition.

To our knowledge, there is one other study that explores the long-term impacts of sugar exposure in early life in a natural experimental setting (34). Exploiting the end of sugar rationing in 1953, they compare UKB participants conceived before to those conceived after and find that exposure to sugar rationing in early life reduces the risk of developing type II diabetes and hypertension. This raises the question of why these results differ. We believe that there are at least two potential reasons for this. First, although both explore the impact of prenatal exposure to sugar, the two experiments are different. In particular, our period of confectionery derationing lasts just under four months, shorter than the length of gestation, meaning that we can identify effects specific to the prenatal period (and even explore possible gestational ages that are sensitive to high exposure). This is not possible in studies that rely on a single discontinuity. The fact that exposed individuals in our sample are *unexposed* for a period of time after birth, restricting their access to sugar, also distinguishes our setting. Hence, our experiment captures short-term prenatal exposure to increased sugar intakes, rather than a continuous increased exposure throughout life. Second, the end of sugar rationing took place in a period that witnessed many other events, making it more challenging to identify their separate contributions to individuals’ outcomes. For example, most other foods were derationed during the early 1950s, and the economy moved from a socialist design to a free-market design, following a right-wing election victory in 1951.

Finally, we explore whether one's genetic “predisposition” exacerbates or alleviates these effects. We find some evidence of genetic heterogeneity of exposure effects. With high polygenic indices for cardiovascular disease and sugar and carbohydrate intake, the beneficial effects of derationing (i.e. of a higher sugar environment in utero) are higher. From a fetal-programming perspective, it seems that a favorable in utero environment is particularly protective for individuals who have a high genetic propensity for adverse late-life outcomes, such as consuming unhealthy foods. This may make sense from an evolutionary point of view: put bluntly, individuals who by their genetic constitution consume little sugar as adults do not need to be prepared by certain early-life exposures that are aimed at influencing sugar consumption. However, there are also alternative explanations. For example, given that the parents of individuals with a high polygenic index for (e.g.) sugar intake are also likely to have a high polygenic index for sugar intake, the G×E estimates may be capturing the additional exposure to confectionery driven by the parental genetic “predisposition” for sugar. This too is consistent with fetal programming, in that the high-sugar environment during the in utero period is better aligned with the later-life environment.

## Materials and methods

### Materials

We use the UKB: a prospective, population-based cohort study of over 500,000 individuals across the United Kingdom born between 1934 and 1971 (14). It has received ethical approval from the NHS North West Centre for Research Ethics Committees (references: 11/NW/0382, 16/NW/0274, 21/NW/0157). We restrict our sample in three ways. First, we consider only those born between 1947 April 24 and 1952 May 13 (i.e. 2 years prior to the start of derationing and 2 years after the last individuals exposed to derationing in utero were born). This restricts the sample to 106,585 individuals. Second, we include only those who are classified as “White British” ancestry. Third, we drop individuals for whom no geographical birth coordinates are available or who were born in Scotland or Northern Ireland.

We select a set of nine outcomes. Because these are all motivated by and well-embedded in the existing literature on the long-term effects of poor prenatal environments (see “Introduction”), we do not apply multiple hypothesis testing. We argue that, given our relatively small (but important) treatment and—with that—likely small downstream effects on our outcomes of interest, there is a nonnegligible chance that multiple hypothesis testing leads to false negatives (type II errors). Furthermore, we cannot assume that our outcomes are independent. For example, increased consumption of sugar is related to a heightened risk of type II diabetes as well as heart disease and obesity. We therefore report our estimates without correcting for multiple testing, but we acknowledge that this is not a black-and-white setting. We follow (35–38) to create a measure of years of education. We also explore effects on birth weight, BMI, (self-reported) type-2 diabetes, and a binary indicator for being diagnosed with cardiovascular disease. The latter is obtained from self-reports (UKB data field 6150) and primary and secondary ICD-10 codes in hospital inpatient records/national death registry. In the records and death registry, we define cardiovascular disease using the ICD-10 codes I (Diseases of the circulatory system) and G45 (Transient cerebral ischemic attacks and related syndromes). Finally, macronutrient intakes represent nutrient *densities*; i.e. the proportion of the total energy intake that is due to sugar, carbohydrates, and fats (39). Appendix C presents descriptive statistics of our analysis sample.

To construct a polygenic index for each of the outcome variables, we run a tailor-made genome-wide association study (GWAS) on UKB participants, excluding our analysis sample to avoid overfitting, and we drop any relatives of UKB participants of third degree and closer to avoid clustering. We use its summary statistics on all single nucleotide polymorphisms to create the relevant polygenic indices for our analysis sample, using LDpred ((40); see Appendix B for more information). All polygenic indices are standardized to have mean zero and SD 1.

### Methods

The baseline specification for the empirical analysis is


(1) $$y_{i}=\alpha_{0}+\;\alpha_{1}E_{i}+\delta X_{i}+f(X_{i},\;E_{i})+\epsilon_{i}$$

where $y_{i}$ is the outcome of interest for individual *i* and $E_{i}$ is a binary indicator equal to 1 if the individual was exposed to at least one day of confectionery derationing while in utero (i.e. who was born between 1949 April 24 and 1950 May 12). In the absence of data on gestational age, we here assume that all pregnancies last 9 months. Hence, our coefficient of interest is $\alpha_{1}$, capturing the effect of prenatal exposure to confectionery derationing on the outcome of interest. Our specification is an “Intention To Treat” (ITT) design since we do not observe individual *consumption* of confectionery and instead estimate the impact of the *policy*. Historical documents and newspaper coverage, however, document that the demand for confectionery rose dramatically during the derationing period compared with the period before. Because of a lack of data on actual confectionery intakes, it is not possible to reliably estimate changes in the prenatal consumption. The National Food Survey, the main UK survey of household food consumption that started in the 1940s, does not record the intake of chocolates and sugar confectionery since it is considered a *personal* rather than *household* good (41).

$X_{i}$ includes gender and 11 binary indicators for the month of birth to remove seasonality in outcomes. It also includes calendar time as a linear function of the year-month of birth (i.e. capturing individuals’ age), as well as the calendar time interacted with the treatment dummy $E_{i}$. We report heteroskedasticity-robust standard errors clustered by year-month of birth. To account for any geographical variation in the nutritional environment during this period, we re-estimate Eq. 1 including region-of-birth fixed effects. At this time, England and Wales were divided into over 60 counties, 230 administrative counties, and 1,400 local government districts (henceforth: districts). Using information on individuals’ location of birth, we assign to each individual a county, administrative county, and district ID and add dummies for each. This allows us to compare individuals in close proximity but at different points in time, some of whom would have been exposed to prenatal derationing and others not. We then cluster standard errors by region of birth, but the results are robust to clustering by year-month of birth instead (Table S6).

The estimates of Eq. 1 capture the average effect of prenatal exposure to confectionery on the outcome of interest. To explore whether those who are genetically “predisposed” to the outcomes are differentially affected, we model the gene–environment interplay in our empirical specification as


(2) $$\begin{array}{rl} y_{i}= & \;\beta_{0}+\beta_{1}G_{i}+\;\beta_{2}E_{i}+\;\beta_{3}G_{i}\times E_{i}+\gamma X_{i}+f_{1}(X_{i},\;E_{i})+f_{2}(X_{i},\;G_{i})\\ & +\overset{10}{\underset{\;p=1}{\sum }}\;\delta_{p}PC_{i}^{p}+e_{i} \end{array}$$

where $G_{i}$ denotes the polygenic index that is specific to outcome $y_{i}$. The right-hand side also includes the first 10 principal components of the genetic data $\sum_{\;p=1}^{10}\delta_{p}{\mathrm{PC}}_{i}^{p}\;$ to account for ancestry (42). Furthermore, we add interactions $f_{1}(X_{i},\;E_{i})$ between the covariates $X_{i}$ and our “exposure indicator” $E_{i}$, and $f_{2}(X_{i},\;G_{i})$ between the covariates and the polygenic index $G_{i}$. This prevents the effects of such interactions from being inadvertently picked up by the estimated effects of the $G_{i}\times E_{i}\;$ interaction (43). In practice, $f_{1}(X_{i},\;E_{i})$ includes the interactions of $E_{i}$ with calendar time, gender, and the principal components, but not with the month fixed effects since its coefficients would partially capture the effect of interest. In contrast, $f_{2}(X_{i},\;G_{i})$ includes the interactions of $G_{i}$ and calendar time, gender, the principal components (PCs), and the month effects.

The coefficients of interest are $\beta_{2}$ and $\beta_{3}$, with the former replacing $\alpha_{1}$ in the baseline specification. The latter captures whether those who are genetically at higher risk of the outcome (whether via a direct genetic effect or via genetic nurture (44, 45)) are more or less likely to be affected by the exposure, compared with those at lower genetic risk.

The ITT design complicates the interpretation of interaction effects. Specifically, for the interaction effect of $G_{i}$ and actual consumption to have the same sign as the interaction effect between $G_{i}$ and the exposure $E_{i}$, the genetic background should not strongly moderate the effect of exposure on consumption. This is likely to be satisfied, e.g. if actual consumption after derationing remains restricted by a limited availability of affordable confectionery, as is the case in our setting. Either way, one might argue that what matters from a policy evaluation perspective are variables that can be directly manipulated, which in our case are the environment indicators (i.e. the rationing of confectionery) rather than individuals’ actual confectionery choices.

## Supplementary Material

pgaf301_Supplementary_Data

## Data Availability

The UK Biobank data are not accessible in the public domain. Researchers can apply for data access directly at http://www.ukbiobank.ac.uk.
