# Supplementary material for: Prenatal sugar exposure shapes late-life human capital and health
Source: PNAS Nexus. 2025 Sep 20;4(10):pgaf301. doi: 10.1093/pnasnexus/pgaf301 (PMC12525142; doi:10.1093/pnasnexus/pgaf301)
Supplement: pgaf301_Supplementary_Data [file pgaf301_supplementary_data.pdf]

Supplementary Material

Prenatal Sugar Exposure Shapes Late-Life Human Capital and Health

Gerard J. van den Berg* Stephanie von Hinke† R. Adele H. Wang‡

*Faculty of Economics and Business, University of Groningen, Groningen, the Netherlands; UMC Groningen; IFAU Uppsala; ZEW; J-PAL and CEPR.

†Corresponding author: School of Economics, University of Bristol, Bristol, United Kingdom; IFS; IZA. E-mail: S.vonHinke@bristol.ac.uk

‡School of Economics, University of Bristol, Bristol, United Kingdom.

We thank the editor Yannis C. Yortsos, two anonymous referees, Andreas Peichl, Fabrice Etilé, Fleur Meddens, Hans van Kippersluis, Niels Rietveld, Pietro Biroli, Rachel Soloveichik and seminar participants at the Erasmus School of Economics, UCL IoE, ISER, UMC Groningen, IFS Nutrition Workshop, RES, CESifo/NORFACE conference and the European Workshop in Econometrics and Health Economics for useful comments and discussions. Samuel Baker, Sean Harrison, and Emil Sørensen provided valuable help with the data. A previous version of this work circulated under the title "Prenatal Sugar Consumption and Late-Life Human Capital and Health: Analyses Based on Postwar Rationing and Polygenic Scores", and was posted on a preprint: https://arxiv.org/abs/2301.09982. This research has been conducted using the UK Biobank Resource under Application Number 74002. We gratefully acknowledge financial support from NORFACE DIAL (Grant Reference 462-16-100), the European Research Council (DONNI 851725) and the EU's HORIZON-MSCA-2021-DN-01 programme (101073237). None of the authors have competing interests.

Supplementary Material

Appendix A The UK rationing system

A.1 The rationing system

Before the Second World War, Britain imported 55m tons of food per year, including 50% of meats, 70-80% of its cheese and sugar, nearly 80% of fruits, about 70% of cereals and fats, and 90% of butter, cereal and flour [1].[1] A month after the war had started, this had dropped to 12m on an annual base.

Five days after the war was declared, in September 1939, individuals received their ration books. Households had to register with a local supplier; the details of which were stamped in the book, as one could only purchase the ration from the registered supplier. The ration books were not used until four months later (on January 8, 1940). The ration coupon was necessary for the individual to get their share of the item. The coupons could not be used on other produce, nor could they be carried forward. As such, rationing was not applied to e.g. seasonal items, as the government was unable to guarantee their year-round supply. Other items that the government did not want to ration include fresh fruit and vegetables, bread, potatoes, cigarettes and beer. One reason was that supplies were limited anyway (e.g., fresh fruit) so the government could not guarantee its availability, or the government wanted to provide sufficient quantities of cheap energy (i.e., bread, potatoes). Another was that rationing these foods would impact morale, which the government wanted to avoid, and – for tobacco – it would have conflicted with its revenue-raising function [2]. In summary, rationing was based on the principle that all essential nutrients, even those in short supply, should be equally available to everyone, to the extent necessary to maintain health, and at controlled prices [3].

With an envisaged significant drop in food imports, the government expected severe food shortages, rising prices, and a growing inability of low SES households to afford food. In addition, food availability was expected to be volatile, leading to hoarding. By analogy to the experiences in Germany towards the end of World War I, food shortages were expected to affect the morale and lead to food riots and civil unrest. One of the main aims of food rationing was therefore to ensure that everyone had access to necessary foods. Incidentally, the UK rationing system did not only cover food but also clothing, fuel, soap, paper, furniture and other products. Furthermore, the rationing system was not only targeted at individual consumers but, depending on the product involved, also restricted inputs of industries, sectors and public services.

To purchase rationed foods, individuals were issued ration books, with different coupons for different foods, e.g., separate coupons for butter, cheese, and sugar. Ration coupons

[1] This Appendix draws heavily on Zweiniger-Bargielowska (2000) and references therein [2]

were the government guarantee that you would be able to get your share of the food, though it could only be used at the shop where the individual had registered, with shop keepers cutting out the relevant coupons when individuals purchased the product. There were different ration books for different population groups. For example, most people received the standard blue ration book, but pregnant women, nursing mothers and children under five received green ones, which entitled them to additional milk, eggs and meat, orange juice, cod liver oil, and vitamin A and D [4].

"Points rationing" was introduced in December 1941 via a separate pink rationing book. This, in conjunction with regular coupon rationing, but was more flexible, giving shoppers greater choice. It could also be used for purchases at grocers other than the one the household had registered with. However, points rationing was mainly used for tinned goods. The Ministry of Food could adjust the price (i.e. the number of points that an item would cost) in order to try to achieve a balance between supply and demand. Prices of unrationed goods were also strictly controlled by the government. As a result, unrationed goods were not always available. Nevertheless, there was a very small black market in luxury goods; a black market in necessities hardly existed [3].

As a secondary aim, the rationing system was used to improve the quality of individuals' nutritional intake and reduce nutritional inequalities between SES groups by steering the accessible food bundle towards a nutritionally healthy diet available to all. The evidence shows that this was successful [3, 5, 6, 7]. Intakes of protein, calcium, iron, vitamin B, riboflavin, vitamin C, and nicotinic acid all increased considerably during wartime rationing. This meant that the average diet of all classes was better balanced than ever before [3]. Indeed, children were taller and heavier than before the war, infant mortality rates reduced and maternal mortality rates dropped [3, 5].

Table S.1 shows a rough timeline of when some of the main foods came on and off rationing. The amount of food that one could get for one coupon tended to vary somewhat over time in line with the quantity of food imports and home-grown produce; see [2] for detailed information.

The years immediately following the end of World War II witnessed a global food crisis, while the UK continued to be heavily dependent on food imports. The war had taken a heavy toll on the UK national debt and the UK insisted on maintaining a large military force abroad after the war. As a result, it faced budgetary and balance of payments difficulties. The postwar socialist government saw food rationing as a policy to limit consumption of imported goods without putting poor households in jeopardy. Hence, it decided to continue the food rationing system and to even further extend the types of food products subject to rationing, ensuring everyone continued to have access to a healthy and balanced diet. This policy continued into the early 1950s when the socialist government lost the national election.

A.2 Rationing of sugar and confectionery

Before the war, about 70% of the sugar used in the UK was imported. Sugar was one of the first batches of products to be rationed in early 1940 and among the last to be derationed in late 1953. Importantly for our purposes, sugar rationing was regarded to be more binding and restrictive than the rationing of most other food products, in the sense that its consumption was reduced more dramatically (see [2], and references therein).

By June 1942, UK food consumption had become heavily dependent on imports from the US and other allies, and food trade was coordinated at the international level. In the first half of 1942, the US lost the Philippines (its prime supplier of sugar) to Japan [8], and import from other areas was hampered by sea warfare. This led to the introduction of sugar rationing in the US, which was followed by the rationing of confectionery products in the UK on July 26, 1942. The latter adopted the "point rationing" system with *individual* (rather than *household*) allowances of ≈3 oz (85g) per person per week. The exact amount varied slightly throughout the rationing years depending on the supply of raw ingredients and special events. Figure S.1 shows that, for example, those under 18 were given an additional 2 oz "Christmas bonus" in December 1944 and December 1946 [9].[2]

As shown in Table S.1, the vast majority of rationing measures was introduced before mid-1942, and most ended in the 1950s.[3] The derationing interval in 1949 did not see large changes to the rationing system, nor did it witness other major societal events.[4] The April 1949 derationing was imposed through an administrative act taken by civil servants in response to pressure by the confectionery industry and an improvement in the supplies of sugar and other ingredients. The industry estimated that the supply of raw materials at the time would allow weekly consumption per capita to rise to 4.75 oz (135g). The trade bodies estimated that demand and supply would find an equilibrium at this level without price adjustments (which would have been politically infeasible;

[2]The confectionery ration coupons could be spent on chocolate and sugar confectionery. Although confectionery and sugar are not identical, confectionery mostly consists of sugar. Additional ingredients may include small amounts of fat, flour and proteins. The two items were consumed in approximately equal proportions in the pre-war years, but the rationing era saw a preference for chocolate with occasional minor surpluses of sugar confectionery, which was subsequently used by the confectionery industry as an argument for the abolishment of rationing in 1949 [10].

[3]Other changes in rationing took place in time periods that witnessed additional upheavals, making it more challenging to identify their separate contributions to health and human capital outcomes. Notably, the onset of rationing coincided with preparations for the war, while the ultimate derationing of confectionery and sugar in 1953 took place in a period in which the rationing of many other products was abolished as well and in which the economy moved from a socialist design to a free-market design following a right-wing election victory.

[4]A notable event close to our derationing interval is the formal establishment of the NHS universal health service in England in July 1948. However, the actual health care system and supply changed rather gradually in this period [11, 12]. Many individuals were insured before July 1948 and aspects of universal care were already available regionally, while after July 1948 certain provisions were in short supply and led to queueing, and other provisions remained costly. The year 1948 also witnessed a gradual tightening of educational requirements for nurses. These overall changes in health care can therefore be accounted for by a flexible trend specification in calendar time in our empirical analysis.

[13]). Importantly, therefore, the decision to deration confectionery was not taken by the cabinet, which could have used it to curry favor with the electorate and which often discussed minor changes in ration levels. Confectionery was completely derationed on April 24, 1949; the announcement was made on February 21, 1949 [13]. To allow traders to build up stock and meet the impact of derationing, civil servants allocated an immediate 50% additional credits to sweets wholesalers and retailers between February and April 1949, plus a further one-off grant of 50% of the coupons collected in March 1949 [14].

As it happened, the actual demand of 8-10 oz (227-283 g) far outstripped supply, with sales in late April 1949 exceeding the levels in April 1948 by 84% [15], leading to long queues at confectionery shops, and a rapid running down of any stocks [2, 16]. By late May 1949, many confectionery shops had run out of stock and closed for business [17]. In a nutshell, the confectionery industry was caught in-between the lack of inputs and the fact that consumer product prices were fixed. In addition, export of confectionery production had priority over domestic consumption [16]. With the Chancellor of the Exchequer announcing a cut in sugar imports for July, the government decided on July 14 to re-introduce confectionery rationing on August 13, 1949; less than four months after the initial derationing. Final derationing of confectionery and sugar only took place in February and September of 1953, respectively, along with the abolishment of rationing of most other products. Subsequent years witnessed steeply increasing consumption.

The above implies that the derationing on April 24, 1949 was exogenous, precisely delineated in time, and difficult to anticipate at the individual level. The reinstallment of rationing was also exogenous at the individual level. However, the point in time at which individuals started to experience confectionery purchase constraints (due to shops running out of stock) most likely took place before the official re-instalment of rationing on August 13, 1949. Therefore we perform a sensitivity analysis, stipulating derationing to end on May 31, 1949.

Table S.1.
Start and end dates of rationing in the UK for selected main foods

	Start	End	Notes
National Registration Day			Sep 29 1939: Provide details of all household members
Gammon, ham	Jan 8, 1940	Oct, 3 1952	
Bacon	Jan 8, 1940	Jul, 3 1954	
Butter	Jan 8, 1940	May, 8 1954	
Sugar	Jan 8, 1940	Sep, 27 1953	
Meat (incl beef, veal, mutton, pork)	Mar, 11 1940	Jul, 4 1954	
Margarine	Jul 1940	May 8, 1954	
Tea	Jul 1940	Oct 5, 1952	
Cooking fat	Jul 1940	May 8, 1954	
Cheese	May 5, 1941	May 1954	Vegetarians were allowed an extra 85g on top of the standard ration
Eggs	Jun 1941	Mar 26, 1953	
Milk	Nov 1941	1950	
Tinned veg, breakfast cereals	Feb 9, 1942	May 19, 1950	Points rationing
Dried and canned fruit, rice	Jan 1942	May 19, 1950	Points rationing
Biscuits	Aug 1942	May 19, 1950	Points rationing
Oats (flaked and rolled)	Dec 1942	May 19, 1950	Points rationing
Syrup, treacle	Jun 1942	May 19, 1950	Transferred from standard rationing to points scheme
Confectionery	Jul 26, 1942	Feb 5, 1953	Temporary derationing between April 24 - August 13 1949

Notes: The precise date of introduction and removal of rationing is not always known.

Figure S.1.
Variation in the sweets ration; 1942 – 1948

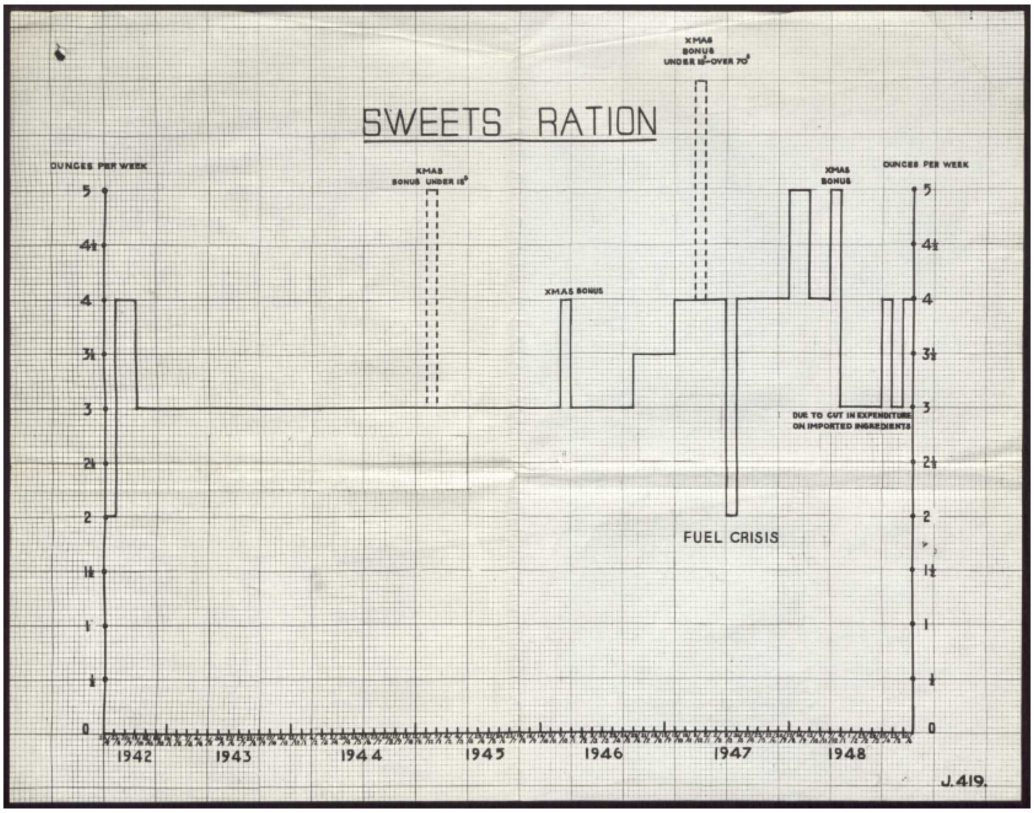

The figure shows the variation in the sweets ration between its introduction on July 26, 1942 and the end of 1948. Source: MAF 156/263, 1951. Crown copyright.

Appendix B Genetic Analysis

B.1 Our tailor-made GWAS

The full UK Biobank data release contains all successfully genotyped samples (n=488,377). Pre-imputation QC, phasing and imputation are described in detail in Bycroft et al. (2018) [18]. In brief, prior to phasing, we removed multiallelic SNPs or those with a minor allele frequency (MAF) $\leq 1\%$. Phasing of genotype data was performed using a modified version of the SHAPEIT2 algorithm [19]. Genotype imputation to a reference set combining the UK10K haplotype and HRC reference panels [20] was performed using IMPUTE2 algorithms [21]. Our analysis is restricted to autosomal variants within the HRC site list using a graded filtering with varying imputation quality for different allele frequency ranges.

We follow the literature and exclude individuals with sex-mismatch (derived by comparing genetic sex and reported sex) or individuals with sex-chromosome aneuploidy from the analysis (n=814). Furthermore, we restrict the sample to individuals of white British ancestry who self-report as 'White British' and who have very similar ancestral backgrounds according to the PCA (n=409,703), as described by [18]. Finally, our GWAS discovery sample only includes unrelated individuals; we exclude genetically related individuals as well as our analysis sample (i.e., those born between 24 April 1947 and 13 May 1952). Using a GWAS sample that is more similar to the analysis sample increases predictive power, since the polygenic index captures the genetic "predisposition" within the environmental context of individuals from the discovery sample [22].

Depending on the distribution of the outcome, we use a linear or logistic regression in the genome-wide association study, using the software PLINKv2.00. We specify the genotype array, gender and the first 10 principal components as covariates.

B.2 Calculating Polygenic Indices

We use LDpred [23] to construct all polygenic indices, including summary statistics for all SNPs from the above GWAS. The Python-based software package adjusts GWAS summary statistics for the effects of linkage disequilibrium (LD). It uses a Bayesian method, assuming a prior for the genetic architecture of the trait of interest in combination with information on the linkage disequilibrium from an external reference panel (30,000 unrelated individuals in UK Biobank) to estimate posterior mean effect sizes from summary statistics. We construct the polygenic indices using an LD window of M/3000 where M is the total number of SNPs. We assume the fraction of SNPs with non-zero effects to be 0.3. Our results are robust to taking a prior of 1 instead of 0.3. We restrict the analysis to 1.2M HapMap 3 SNPs [24] to ensure a set of common, well-imputed variants.

Appendix C Descriptive Statistics

The UK Biobank is a prospective, population-based cohort study that contains data on the health and well-being of over 500,000 individuals across the United Kingdom, born between 1938–1971. Recruitment and collection of baseline data took place between 2006 and 2010, when participants were aged between 40–69. Individuals provided information on demographics, health status, lifestyle measures, cognitive testing, personality self-report, and physical and mental health measures via questionnaires and interviews. Furthermore, anthropometric measures were taken by nurses, along with blood pressure readings and samples of blood, urine and saliva. All individuals have been genotyped. The sample is not representative of the UK population, with UK Biobank participants more likely to live in urban areas and be of higher socio-economic status (Fry et al., 2017). There is no evidence, however, of additional sample selection: using the UK Biobank sample, we find no evidence of differences in the number of births or in its trend during the confectionery derationing period.

Table S.2 presents the descriptive statistics of the outcomes of interest, including the maximum sample sizes for the analysis sample of each outcome. This shows that, on average, UKB participants born between April 1947 and May 1952 have over 14 years of education, a birth weight of 3332g and a BMI of 27.6. Approximately 51% of the sample has been diagnosed with any form of cardiovascular disease (note this includes self-reported hypertension; see Section **??**) and 5% have been diagnosed with type-2 diabetes. Approximately 23% of individuals' total energy intake is derived from sugar, 48% from carbohydrates and 33% from fats. Just under half of the sample is male.

TABLE S.2.
Descriptives

	N	mean	sd
Years of education	84,165	14.82	(5.118)
Birth weight (in g)	47,452	3331.9	(672.2)
BMI	84,513	27.58	(4.795)
Height (in cm)	84,600	168.51	(9.220)
Cardiovascular disease	84,631	0.51	(0.499)
Type-2 diabetes	84,596	0.05	(0.222)
Sugar intake	36,127	0.23	(0.069)
Carbohydrate intake	36,127	0.48	(0.082)
Fat intake	36,127	0.33	(0.067)
Male	84,784	0.45	(0.498)

Sample sizes, means and standard deviations of the estimation sample, including individuals born between April 1947 (i.e., two years prior to the temporary sugar derationing) and May 1952 (i.e., two years and nine months post the end of the temporary sugar derationing). Variable definitions are given in Section **??**.

Figure S.2 presents the trends in each outcome variable for our period of observation by individuals' year-month of birth, where the two vertical lines indicate the first and last

cohort exposed prenatally to the temporary confectionery derationing period. The figures suggest a slight reduction in BMI and sugar intakes for those prenatally exposed to the derationing. However, there is substantial variation in each of the outcome variables.

Figure S.2.
Trends in the outcomes of interest

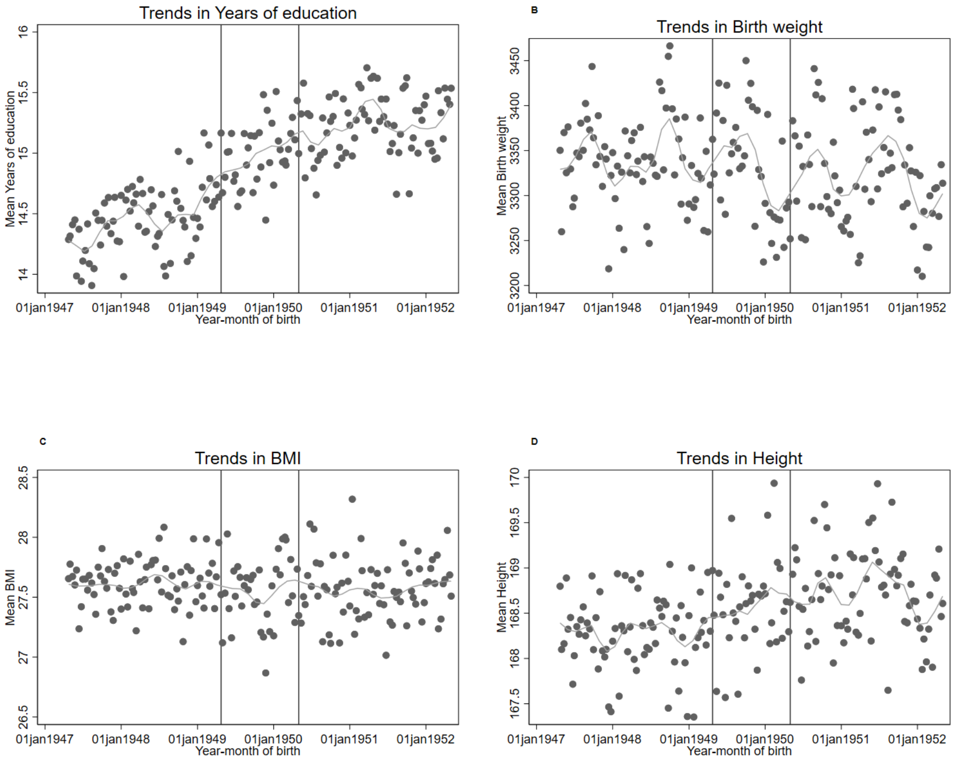

To gauge evidence for gene-environment correlations (rGE), Figure S.3 in Appendix C plots the densities of each of the (standardized) polygenic indices for those exposed to derationing in utero (solid line) and those unexposed (dashed line). From the very similar polygenic index distributions we conclude that there is little evidence of polygenic indices being correlated with the exposure indicator. The scatter plots overlaying the densities are the averages of each outcome for 200 equal sized bins of the corresponding polygenic index. The fitted curve through the dots is obtained from a kernel-weighted local polynomial regression of the outcome on the corresponding polygenic index, where we again distinguish between the exposed and unexposed groups. This shows a strong correlation between the polygenic index and each of the corresponding outcomes. The statistical strength of this relationship is investigated in Section ??. Finally, the figures suggest that we can approximate the relationship between the polygenic index and the respective outcome as linear.

Trends in the outcomes of interest (cont.)

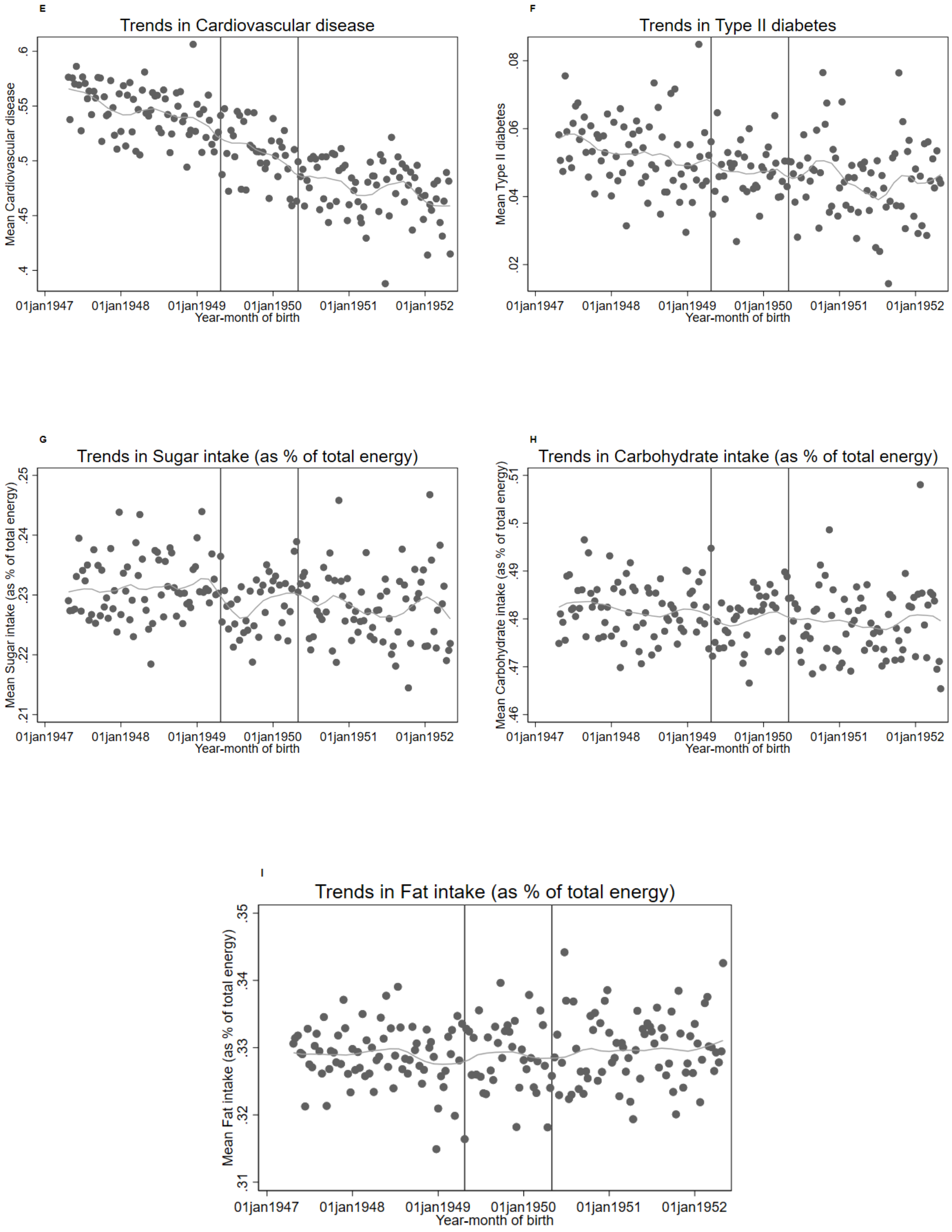

Notes: The figures plot the trends of the relevant outcome variable by year-month of birth, where we separate each month into "early" (born prior to the 12th of the month), "mid" (between the 13th and 23rd of the month) and "late" (born on the 24th of the month or after). Hence, we plot three observations for each month. The exposed cohorts are born between April 24, 1949 and May 12, 1950 (i.e., 9 months (minus one day) after the reintroduction of rationing on August 13, 1949). CVD denotes cardiovascular disease; T2D denotes Type II Diabetes; Nutritional intakes are measured as a percent of total energy.

FIGURE S.3.
Gene-environment correlations

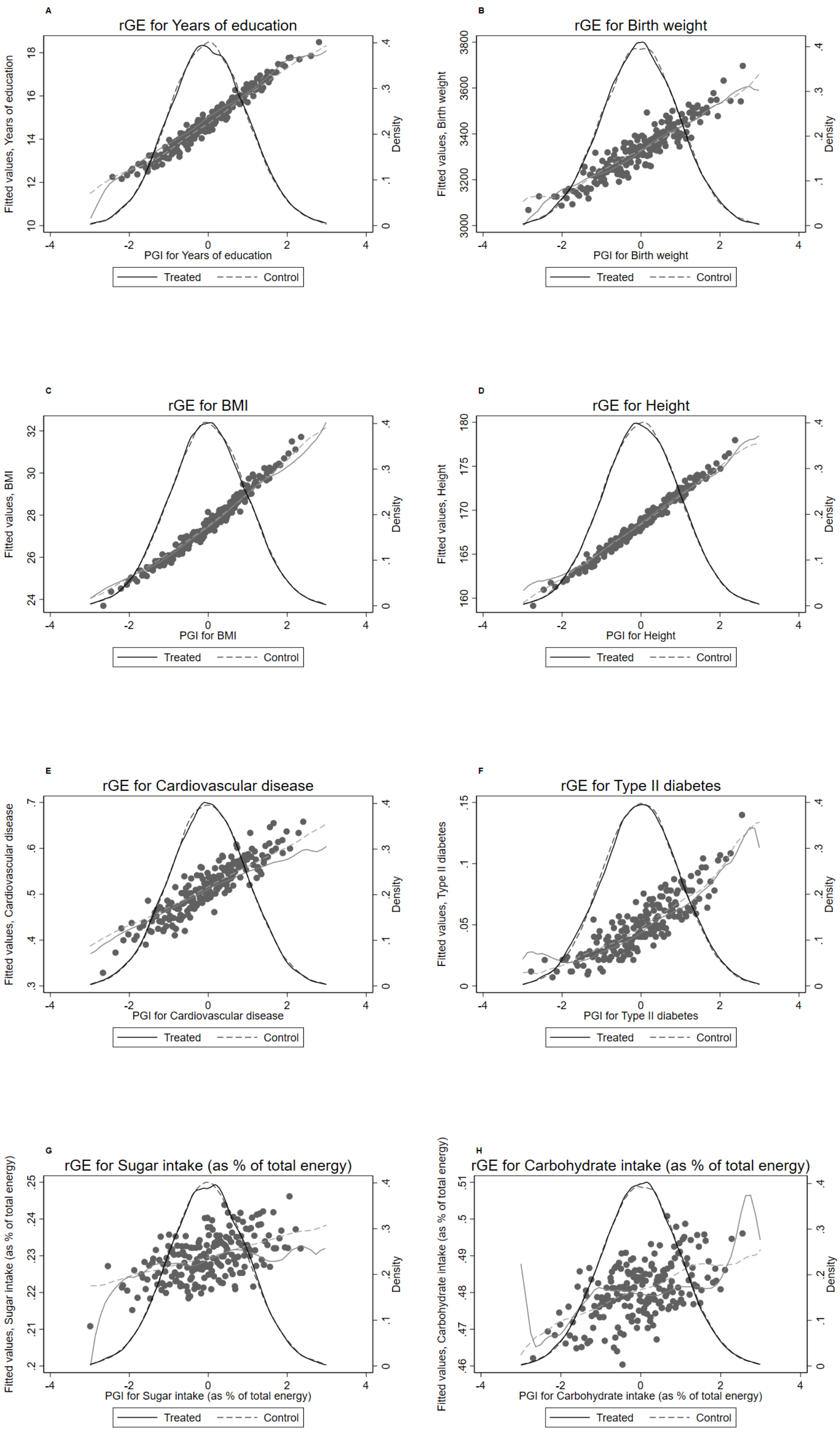

Notes: The figures plot the distribution of the different polygenic indices by treatment status. The treated are defined as those exposed to at least one day of confectionery derationing whilst in utero (i.e., born between April 24, 1949 and May 12, 1950). The controls are defined as those born just before or after this period. The full analysis sample only includes individuals born between April 24, 1947 and May 13, 1952. The graphs show the predictive power of the polygenic index (created using our tailor-made GWAS on the non-sibling sample of the UK Biobank, excluding the estimation sample) for their respective outcomes. CVD denotes cardiovascular disease; T2D denotes Type II Diabetes; Nutritional intakes are measured as a percent of total energy.

Appendix D Additional Tables and Figures

The following subsections discuss a series of additional analyses and sensitivity checks to ensure that our analysis is robust to different assumptions. This includes an analysis of trimester-specific effects, sensitivity to the timing of the derationing period, the role of the length of time exposed in utero, accounting for potential selection into pregnancy, and alternative specifications of the calender-time trend covariate. We also run a set of placebo checks and explore possible gender differences in the treatment effect. For each of these, we focus on the main effect of exposure to the derationing, rather than the $G \times E$ effects (the latter are available upon request). All analyses control for administrative county fixed effects, unless otherwise stated.

D.1 Trimester-specific exposure

Our baseline analysis above estimates effects of being exposed to confectionery derationing averaged over all those in utero during this period. However, the literature on the long-term effects of intrauterine exposures highlights potentially heterogeneous effects due to exposure at different stages of the pregnancy, generally distinguishing between three trimesters [25, 26]. For example, most organs are formed during the first trimester, so adverse circumstances in this period may primarily affect organ development.

To explore whether there are differential effects of exposure by trimester, we extend the baseline specification by allowing for three separate "trimester effects". This requires a mapping from the actual pregnancy interval into the gestational trimester j of exposure. In this we follow the literature on the effects of the Dutch Hunger Winter famine which also lasted about 4 months [25]. In equation (??), we replace $\alpha_1 E_i$ by $\sum_{j=1}^{3} \alpha_j E_{ij}$, where each E_{ij} is binary and $\sum_{j=1}^{3} E_{ij} \leq 1$.[5] The results are presented in Panel A of Table S.1. This shows that the positive effect on years of education is largely driven by exposure in the third trimester, with this leading to an increase of 0.2 years of schooling, with a negative (insignificant) effect for first trimester exposure. The (marginally significant) positive effect on birth weight is driven by exposure in the first and particularly second trimester. Finally, the negative effect on BMI is mainly driven by exposure during the second and third trimester, and the negative effect on sugar consumption is similar across the three trimesters.

During the third trimester, the fetus gains most weight, so malnutrition in this trimester may cause the caloric needs to support intrauterine growth not to be fulfilled. Conditions in the UK in 1949 cannot be accurately described as malnutrition. However, appetite may depend on the type of food. Put simply, in the third trimester it may be

[5] Exposure in the first trimester is defined as one for those born between December 24, 1949 and May 12, 1950, and zero otherwise. Those born between September 24, 1949 and December 23, 1949 are defined as being exposed in the second trimester, and those born between April 24, 1949 and September 23, 1949 are defined as being exposed in the third trimester.

more convenient to consume confectionery than to eat a huge plate of cooked potatoes. In settings where caloric supply is sufficient, such considerations may be relevant and may have long-run implications, which may explain some of the trimester-specific results.

D.2 Timing of confectionery derationing

Although confectionery derationing started on April 24, 1949 and was abolished on August 13, 1949, its effects on actual consumption may have been restricted to a narrower time interval. As discussed earlier in the paper, the demand for confectionery was so high that there were long queues outside shops, with evidence that, by May 1949, some confectionery shops had run out of stock. This would imply that some individuals who are currently defined as treated are in fact not exposed to confectionery prenatally due to a non-availability, potentially attenuating our estimate of interest. To explore this in more detail, we re-run the analysis, setting E_i equal to one for individuals who were in utero between April 24, 1949 and the end of *May 1949* (instead of August 13, 1949). The estimates are presented in Panel B of Table S.1. These confirm the positive effect of derationing exposure on years of education and birth weight. Furthermore, we again find that prenatal exposure to derationing led to a drop in BMI of 0.08 units (equivalent to a reduction in body weight of 0.23 kg for a person of average height) and a 0.2 percentage point drop in the sugar density of the diet. All these results are consistent with those found for the baseline specification.

In Panel C, we additionally report the trimester-specific effects when exposure is defined as having been in utero between April 24, 1949 and the end of May 1949.[6] The findings are broadly consistent with those above, with second trimester exposure for birth weight being most important, third trimester exposure for BMI, and first trimester exposure for height. We also find a significant effect for years of education of second trimester exposure, which is also driving the effect on sugar intake.

D.3 The length of time exposed in utero

Our main specification specifies the outcomes as a function of a binary indicator of whether the individual was exposed to confectionery derationing in utero. We next use an empirical specification that allows for what one, with some abuse of language, could call a "dose-response" relationship, allowing those exposed to confectionery derationing for a longer time period to have been impacted more. Specifically, we use the count of the number of months the individual was gestationally exposed, ranging from 1–4, as the

[6]First trimester exposure is now defined as those born between November 13, 1949 and February 28, 1950. Those born between August 13, 1949 and November 12, 1949 are defined as being exposed in the second trimester, and those born between April 24, 1949 and August 12, 1949 are defined as being exposed in the third trimester.

variable of interest.[7]

Table S.2 presents the results, showing that each additional month of exposure increases education by 0.055 years. Similarly, we find a 6g increase in birth weight, a small reduction in BMI of 0.02–0.03 units for each month of exposure, as well as a drop in sugar (and carbohydrate) intakes of 0.01 percentage points per month of exposure. These results are consistent with the findings above and suggest that longer in utero exposure to the confectionery derationing led to larger effects on the outcomes of interest. We again find no statistically significant effects on height, cardiovascular disease, type-2 diabetes and fat intake.

D.4 Timing of pregnancy

We next explore the assumption that pregnancies are not timed relative to the derationing period. For this we exclude individuals who (we infer) were not yet conceived at the time of the government *announcement* (as opposed to *implementation*) of confectionery derationing on 21 February 1949 but who were conceived before the end of derationing. In effect, we compare pregnancies which were unexposed to derationing (including all those born before April 24, 1949) to those who were already pregnant when the government announced the policy, removing any potential selection into pregnancy due to the announced derationing. The latter group now includes those born between April 24, 1949 and November 24, 1949 (i.e., 9 months after the announcement on 21 February 1949). Table S.3 presents the estimates, showing a positive effect on education and a negative effect on BMI and sugar intake. The magnitude of the effects is very similar to those shown above, suggesting there is no selection into pregnancy after the government announcement to deration confectionery.

D.5 Alternative functional forms for calendar-time trend effects

With a relatively small observation window (those born between April 24, 1947 and May 24, 1952), most outcomes do not display strong non-linear trends in birth date among the non-exposed, suggesting that linear specifications in the year-month of birth are sufficient. We test the robustness of this in Table S.4. Here, Panel A only accounts for a linear function in the year-month of birth (i.e., not allowing this to differ by exposure status). In Panel B a quadratic function in the year-month of birth is assumed whereas in Panel C we allow for differential quadratic functions by exposure status. Finally, Panel

[7]More specifically, those born between April 24, 1949 and May 23, 1949, as well as those born between April 14, 1950 and May 13, 1950 are defined as being exposed for one month (i.e., either the last month of pregnancy or the first). Those born between May 24, 1949 and June 23, 1949, as well as those born between March 14, 1950 and April 13, 1950 are defined as being exposed for two months. Those born between June 24, 1949 and July 23, 1949, as well as those born between February 14, 1950 and March 13, 1950 are defined as being exposed for three months. All others born within the relevant time period are defined as being exposed for four months.

D accounts for administrative county-specific annual trends (as well as administrative county fixed effects) to allow for differential trends in food availability at the local level. The results are generally consistent to those above: being exposed to the derationing in utero increases individuals' years of schooling, with some evidence that it reduces BMI and sugar consumption later in life. Although the magnitude of the point estimates are similar to those above, they are not always statistically significantly different from zero. The exception is birth weight, for which the estimates are less robust to the different functional form specifications.

D.6 Placebo analyses

To ensure that our analyses capture the impact of in utero exposure to confectionery derationing rather than other confounding factors, we run three placebo (or negative control) analyses. First, we incorrectly specify the confectionery derationing period to be five years later, from 24/4/1954 to 13/8/1954. Similar to the main analysis, we restrict the estimation sample to include those born two years prior to the start of this 'placebo' derationing period and two years after the last individuals exposed to the 'placebo' derationing in utero were born (i.e., those born between 24/4/1952 and 12/5/1947). The results are presented in Panel A of Table S.7 showing no evidence that the placebo treatment affects our outcomes of interest.

Note that the treated and control groups in Panel A include some individuals who were exposed to the actual sugar (and confectionery) rationing that was in place until September 1953. We therefore repeat these analyses but exclude all those prenatally exposed to actual sugar rationing. In other words, we drop those born before 24/5/1954 (i.e., 9 months after the end of sugar rationing in September 1953), so our sample covers only those born between 24/5/1954 and 12/5/1957. The estimates are reported in Panel B of Table S.7, again showing no impacts on any of our outcomes of interest.

Third, we run our analyses but define the outcome of interest as two variables that arguably cannot be affected by confectionery consumption: individuals' eastings and northings at birth. We run the same analysis as above, but set these as the outcomes of interest and present the results in Table S.8. As expected, this shows no consistent evidence that prenatal exposure to confectionery derationing affects one's eastings and northings of birth. We therefore conclude that the negative control analyses support our main conclusions.

D.7 Heterogeneous effects

Finally, we explore heterogeneity of the treatment effects with respect to gender. Male fetuses are known to be more vulnerable than female fetuses, with the latter being more likely to survive adverse intrauterine conditions (see e.g., Kraemer, 2000). As the dera-

tioning is likely to have led to a relatively mild change in the prenatal environment, it is unlikely to cause differential survival between the genders. Table S.5 reports estimates separately for women (Panel A) and men (Panel B). This shows that although both genders show a positive effect on years of education, it is larger and only significant for men, whilst the positive effect on birth weight is driven by women. We find a reduction in BMI for both genders, but it is only significantly different from zero for women. Finally, although the negative effect on the share of energy from sugar is found for both genders, it is larger and significantly different from zero only for men.

Table S.1.
Timing of derationing and trimester-specific results

	(1) Years of Education	(2) Birth weight (in g)	(3) BMI	(4) Height (in cm)	(5) CVD	(6) Type II Diabetes	(7) Sugar intake	(8) Carb intake	(9) Fats intake
**Panel A: By trimester**									
E: *In utero* during trimester 1	-0.099	47.821	0.133	0.234	0.024	0.001	-0.003	-0.005	0.004
	(0.219)	(35.995)	(0.174)	(0.278)	(0.021)	(0.011)	(0.005)	(0.006)	(0.005)
E: *In utero* during trimester 2	0.097	62.941***	-0.169	0.073	0.005	-0.005	-0.003	-0.005	0.002
	(0.117)	(21.256)	(0.123)	(0.167)	(0.013)	(0.005)	(0.003)	(0.004)	(0.003)
E: *In utero* during trimester 3	0.173**	-1.350	-0.080	-0.051	-0.007	-0.002	-0.002	-0.000	-0.002*
	(0.074)	(12.487)	(0.061)	(0.085)	(0.008)	(0.003)	(0.001)	(0.002)	(0.001)
**Panel B: De-rationing until May '49**									
E: *In utero* during de-rationing	0.138***	17.331*	-0.080*	0.035	-0.000	-0.002	-0.002*	-0.001	-0.001
	(0.044)	(8.906)	(0.041)	(0.061)	(0.006)	(0.002)	(0.001)	(0.001)	(0.001)
**Panel C: De-rationing until May '49**									
E: *In utero* during trimester 1	0.196*	19.947	-0.111	0.245**	0.008	-0.001	-0.001	0.003	-0.001
	(0.108)	(16.308)	(0.083)	(0.107)	(0.009)	(0.004)	(0.003)	(0.003)	(0.002)
E: *In utero* during trimester 2	0.191**	23.526*	-0.023	-0.152	0.005	-0.002	-0.004**	-0.003	0.002
	(0.095)	(13.678)	(0.081)	(0.109)	(0.010)	(0.004)	(0.002)	(0.002)	(0.002)
E: *In utero* during trimester 3	0.073	12.221	-0.097*	0.025	-0.008	-0.003	-0.001	-0.002	-0.002*
	(0.082)	(16.998)	(0.056)	(0.091)	(0.009)	(0.003)	(0.001)	(0.002)	(0.001)
No. of observations	84165	47452	84513	84600	84631	84596	36127	36127	36127

Estimates from Equation (**??**). Panel A reports the estimates of exposure to derationing on the different outcomes of interest, distinguishing by the trimester of exposure. Exposure in the first trimester is defined as those born between December 24, 1949 and May 12, 1950. Those born between September 24, 1949 and December 23, 1949 are exposed in the second trimester, and those born between April 24, 1949 and September 23, 1949 are exposed in the third trimester. Panel B defines the derationing period to be between 24 April 1949 and 31 May 1949, following the literature that suggests that many shops had run out of confectionery by then. Panel C distinguishes between trimesters of exposure within this shorter period of derationing. First trimester exposure is here defined as those born between November 13, 1949 and February 28, 1950. Those born between August 13, 1949 and November 12, 1949 are exposed in the second trimester, and those born between April 24, 1949 and August 12, 1949 are exposed in the third trimester. All regressions include administrative county of birth fixed effects. CVD denotes cardiovascular disease. Robust standard errors, clustered by administrative county of birth, in parentheses. * $p < 0.10$, ** $p < 0.05$, *** $p < 0.01$.

Table S.2.
Role of the number of months exposed to confectionery derationing

	(1) Years of Education	(2) Birth weight (in g)	(3) BMI	(4) Height (in cm)	(5) CVD	(6) Type II Diabetes	(7) Sugar intake	(8) Carb intake	(9) Fats intake
**Panel A: No area fixed effects**									
No. of months exposed to derationing in utero	0.055***	5.853*	-0.026	-0.009	-0.001	-0.001	-0.001***	-0.001*	-0.000
	(0.016)	(3.449)	(0.016)	(0.023)	(0.002)	(0.001)	(0.000)	(0.000)	(0.000)
**Panel B: Admin. county FE**									
No. of months exposed to derationing in utero	0.053**	6.197*	-0.022	-0.014	-0.001	-0.001	-0.001***	-0.001*	-0.000
	(0.021)	(3.738)	(0.017)	(0.022)	(0.002)	(0.001)	(0.000)	(0.000)	(0.000)
Mean of outcome	14.82	3331.90	27.58	168.51	0.51	0.05	0.23	0.48	0.33
No. of observations	84165	47452	84513	84600	84631	84596	36127	36127	36127

Estimates from Equation (**??**), replacing the treatment dummy (E) with a variable specifying the number of months that the individual was exposed to confectionery derationing, ranging from 1–4. CVD denotes cardiovascular disease. Robust standard errors (clustered by year-month in Panel A and by administrative county of birth in Panel B), in parentheses. * $p < 0.10$, ** $p < 0.05$, *** $p < 0.01$.

Table S.3.
Only including those already in utero at the time of the derationing announcement

	(1) Years of Education	(2) Birth weight (in g)	(3) BMI	(4) Height (in cm)	(5) CVD	(6) Type II Diabetes	(7) Sugar intake	(8) Carb intake	(9) Fats intake
**Panel A: No area fixed effects**									
E: *In utero* during derationing	0.152***	14.286	-0.098*	-0.046	-0.003	-0.003	-0.002*	-0.002	-0.001
	(0.050)	(10.626)	(0.057)	(0.074)	(0.007)	(0.002)	(0.001)	(0.001)	(0.001)
**Panel B: Admin. county FE**									
E: *In utero* during derationing	0.129**	14.969	-0.085*	-0.062	-0.002	-0.003	-0.002**	-0.002	-0.001
	(0.063)	(12.095)	(0.047)	(0.075)	(0.007)	(0.002)	(0.001)	(0.001)	(0.001)
Mean of outcome	14.79	3336.10	27.58	168.49	0.52	0.05	0.23	0.48	0.33
No. of observations	76754	43211	77073	77154	77189	77159	32929	32929	32929

Sample includes those born between April 1947 and 24 November 1949 (i.e., 9 months post the announcement of derationing), and between 13 May 1950 (i.e., 9 months after re-rationing) and 12 May 1952, with those born between 24 April 1949 and 24 November 1949 defined as treated. Panel A does not control for region of birth fixed effects; Panel B controls for administrative county fixed effects. CVD denotes cardiovascular disease. Robust standard errors, clustered by year-month of birth in Panel A and by administrative county of birth in Panel B, in parentheses. * $p < 0.10$, ** $p < 0.05$, *** $p < 0.01$.

Table S.4.
Robustness analysis - allowing for different specifications of the calendar time trend effect

	(1) Years of Education	(2) Birth weight (in g)	(3) BMI	(4) Height (in cm)	(5) CVD	(6) Type II Diabetes	(7) Sugar intake	(8) Carb intake	(9) Fats intake
**Panel A**									
E: *In utero* during derationing	0.134***	-0.779	-0.062	0.096	-0.002	-0.002	-0.001	-0.001	-0.001
	(0.038)	(8.464)	(0.039)	(0.059)	(0.005)	(0.002)	(0.001)	(0.001)	(0.001)
**Panel B**									
E: *In utero* during derationing	0.100**	-6.188	-0.070	0.080	-0.002	-0.001	-0.002	-0.000	-0.001
	(0.049)	(10.043)	(0.052)	(0.071)	(0.006)	(0.002)	(0.001)	(0.001)	(0.001)
**Panel C**									
E: *In utero* during derationing	0.134**	14.807	-0.092	0.000	-0.001	-0.002	-0.003***	-0.002	0.000
	(0.057)	(11.355)	(0.058)	(0.074)	(0.008)	(0.003)	(0.001)	(0.001)	(0.001)
**Panel D**									
E: *In utero* during derationing	0.136***	15.804	-0.086**	-0.001	-0.002	-0.003	-0.002*	-0.002	-0.001
	(0.050)	(10.256)	(0.042)	(0.066)	(0.006)	(0.002)	(0.001)	(0.001)	(0.001)
Mean of outcome	14.82	3331.90	27.58	168.51	0.51	0.05	0.23	0.48	0.33
No. of observations	84165	47452	84513	84600	84631	84596	36127	36127	36127

Estimates from Equation (**??**), with the treatment dummy (E) specified as those born between 24 April 1949 and 12 May 1950. Panel A accounts for a linear function in the year-month of birth; Panel B allows for a quadratic function in the year-month of birth; Panel C allows for different quadratic functions for exposed and non-exposed. Panel D accounts for administrative county-specific annual trends (and fixed effects). All regressions control for administrative-county-of-birth fixed effects. CVD denotes cardiovascular disease. Robust standard errors, clustered by administrative county, in parentheses. * $p < 0.10$, ** $p < 0.05$, *** $p < 0.01$.

Table S.5.
Gender differences

	(1) Years of Education	(2) Birth weight (in g)	(3) BMI	(4) Height (in cm)	(5) CVD	(6) Type II Diabetes	(7) Sugar intake	(8) Carb intake	(9) Fats intake
**Panel A: Women**									
D: *In utero* during de-rationing	0.096	19.085	-0.105	0.000	0.004	-0.002	-0.001	-0.001	-0.001
	(0.070)	(11.582)	(0.064)	(0.087)	(0.009)	(0.002)	(0.001)	(0.002)	(0.001)
Mean of outcome	14.29	3247.08	27.26	162.40	0.46	0.03	0.24	0.49	0.33
No. of observations	45982	29009	46188	46230	46231	46239	19015	19015	19015
**Panel B: Men**									
D: *In utero* during de-rationing	0.192**	0.648	-0.050	-0.001	-0.008	-0.004	-0.003*	-0.002	-0.001
	(0.084)	(14.360)	(0.063)	(0.093)	(0.008)	(0.004)	(0.001)	(0.002)	(0.002)
Mean of outcome	15.46	3465.32	27.97	175.86	0.58	0.07	0.22	0.47	0.33
No. of observations	38183	18443	38325	38370	38400	38357	17112	17112	17112

Estimates from Equation (**??**), with the treatment dummy (E) specified as those born between 24 April 1949 and 12 May 1950. Panel A shows the results for women; Panel B shows the results for men. All regressions include administrative county of birth fixed effects. CVD denotes cardiovascular disease. Robust standard errors, clustered by adminsitrative county of birth, in parentheses. * $p < 0.10$, ** $p < 0.05$, *** $p < 0.01$.

Table S.6.
Effects of prenatal exposure to confectionery derationing: Clustering the standard errors by year-month in all panels

	(1) Years of Education	(2) Birth weight (in g)	(3) BMI	(4) Height (in cm)	(5) CVD	(6) Type II Diabetes	(7) Sugar intake	(8) Carb intake	(9) Fats intake
**Panel A: No area fixed effects**									
E: *In utero* during derationing	0.157***	14.918	-0.095*	0.013	-0.003	-0.003	-0.002*	-0.002	-0.001
	(0.047)	(9.608)	(0.051)	(0.066)	(0.006)	(0.002)	(0.001)	(0.001)	(0.001)
**Panel B: County FE (n=65)**									
E: *In utero* during derationing	0.144**	15.599*	-0.089*	-0.001	-0.002	-0.003	-0.002*	-0.001	-0.001
	(0.056)	(9.350)	(0.050)	(0.066)	(0.005)	(0.002)	(0.001)	(0.001)	(0.001)
**Panel C: Admin. county FE (n=232)**									
E: *In utero* during derationing	0.138***	15.422*	-0.083*	-0.005	-0.002	-0.003	-0.002*	-0.002	-0.001
	(0.052)	(9.270)	(0.048)	(0.064)	(0.005)	(0.002)	(0.001)	(0.001)	(0.001)
**Panel D: District FE (n=1434)**									
E: *In utero* during derationing	0.137***	14.708	-0.087*	0.000	-0.002	-0.003	-0.002**	-0.002	-0.001
	(0.051)	(9.557)	(0.049)	(0.065)	(0.005)	(0.002)	(0.001)	(0.001)	(0.001)
Mean of outcome	14.82	3331.90	27.58	168.51	0.51	0.05	0.23	0.48	0.33
No. of observations	84165	47452	84513	84600	84631	84596	36127	36127	36127

Estimates from Equation (??), with the treatment dummy (E) specified as those born between 24 April 1949 and 12 May 1950. All panels report robust standard errors clustered by year-month in parentheses. * $p < 0.10$, ** $p < 0.05$, *** $p < 0.01$.

Table S.7.
Effects of prenatal exposure to confectionery derationing: Negative control, setting the derationing period five years later

	(1) Years of Education	(2) Birth weight (in g)	(3) BMI	(4) Height (in cm)	(5) CVD	(6) Type II Diabetes	(7) Sugar intake	(8) Carb intake	(9) Fats intake
**Panel A: 24/4/1952 – 12/5/1957**									
E: *In utero* during derationing	0.055	10.122	0.025	-0.013	-0.003	-0.001	-0.001	-0.002	0.001
	(0.043)	(8.320)	(0.049)	(0.063)	(0.005)	(0.002)	(0.001)	(0.001)	(0.001)
**Panel B: 24/5/1954 – 12/5/1957**									
E: *In utero* during derationing	0.050	7.225	0.041	0.033	-0.000	0.002	0.002	0.000	-0.003
	(0.111)	(16.689)	(0.110)	(0.154)	(0.012)	(0.005)	(0.002)	(0.003)	(0.002)
Mean of outcome	15.64	3306.94	27.46	169.13	0.39	0.04	0.23	0.48	0.33
No. of observations	34925	22825	35003	35046	35059	35048	15079	15079	15079

Estimates from Equation (??), with the treatment dummy (E) specified as those born between 24 April 1954 and 12 May 1955. All analyses control for administrative county fixed effects. Panel A includes those born between 24/4/1952 – 12/5/1957. Panel B includes those born between 24/5/1954 – 12/5/1957. CVD denotes cardiovascular disease. All panels report robust standard errors clustered by year-month in parentheses. * $p < 0.10$, ** $p < 0.05$, *** $p < 0.01$.

Table S.8.

Effects of prenatal exposure to confectionery derationing: Negative control, specifying two outcomes (eastings and northings of birth) that should not be affected by prenatal derationing

	(1) Eastings of birth	(2) Northings of birth
**Panel A: No area fixed effects**		
E: *In utero* during derationing	543.0	-416.7
	(996.2)	(1140.6)
**Panel B: Administrative county FE (n=232)**		
E: *In utero* during derationing	-44.3	188.1
	(141.5)	(127.8)
Mean of outcome	426162.2	328095.8
No. of observations	84762	84762

Estimates from Equation (**??**), with the treatment dummy (E) specified as those born between 24 April 1949 and 12 May 1950. All panels report robust standard errors clustered by year-month in parentheses. * $p < 0.10$, ** $p < 0.05$, *** $p < 0.01$.

References

[1] Ministry of Food. *The urban working-class household Diet: 1940-1949.* HM Stationery Office, 1951.

[2] Ina Zweiniger-Bargielowska. *Austerity in Britain: Rationing, controls, and consumption, 1939-1955.* OUP Oxford, 2000.

[3] Ministry of Health. On the state of the public health during six years of war, 1946.

[4] Norman Davies. *Europe: A history.* Random House, 2014.

[5] John Burnett. *Plenty and want: A social history of food in England from 1815 to the present day.* Routledge, 2013.

[6] Department of Health. Scientific review of the welfare food scheme. *Reports on health and social subjects*, (51):i–147, 2002.

[7] Shayla Goldring, Nigel Henretty, Julie Mills, Kate Johnson, and Steve Smallwood. Mortality of the 'Golden Generation': What can the ONS Longitudinal Study tell us? *Population Trends*, 145:203–232, 2011.

[8] Richard B Keeton. Price ceilings and rationing: The base ingredients of the black market food industry in Nevada during World War II. *Psi Sigma Siren*, 7(1):6, 2011.

[9] MAF 156/262. Rationing statistics: Straight rationing (part i), 1946-1947. *The National Archives*, 1947.

[10] Financial Times. Changes in the confectionery industry: Complete derationing urged by manufacturers. (Tuesday 15 February 1949), 1949.

[11] G Rivett. From cradle to grave: The history of the NHS 1948-1987. *Nuffield Trust, London*, 2014.

[12] M Thomson. The birth pains of the NHS: How Britain's National Health Service was created. *History Magazine, University of Warwick*, 2018.

[13] Financial Times. All sweets freed. (Tuesday 22 February 1949), 1949.

[14] MAF 156/270. WWII National Food Survey, 1952. cabinet and general monthly report: Points rationing and welfare foods, 1947-1952. *The National Archives*, 1952.

[15] Financial Times. Retail trade up in April. (Saturday 11 June 1949), 1949.

[16] D Royle. Sweet rationing in World War Two. *Working paper, Sawbridgeworth Local History Society*, 2020.

[17] Robert Fitzgerald. *Rowntree and the marketing revolution, 1862-1969.* Cambridge University Press, 1995.

[18] Clare Bycroft, Colin Freeman, Desislava Petkova, Gavin Band, Lloyd T Elliott, Kevin Sharp, Allan Motyer, Damjan Vukcevic, Olivier Delaneau, Jared O'Connell, et al. The UK Biobank resource with deep phenotyping and genomic data. *Nature*, 562(7726):203–209, 2018.

[19] Jared O'Connell, Kevin Sharp, Nick Shrine, Louise Wain, Ian Hall, Martin Tobin, Jean-Francois Zagury, Olivier Delaneau, and Jonathan Marchini. Haplotype estimation for biobank-scale data sets. *Nature Genetics*, 48(7):817–820, 2016.

[20] Jie Huang, Bryan Howie, Shane McCarthy, Yasin Memari, Klaudia Walter, Josine L Min, Petr Danecek, Giovanni Malerba, Elisabetta Trabetti, Hou-Feng Zheng, et al. Improved imputation of low-frequency and rare variants using the UK10K haplotype reference panel. *Nature Communications*, 6(1):8111, 2015.

[21] Bryan Howie, Jonathan Marchini, and Matthew Stephens. Genotype imputation with thousands of genomes. *G3: Genes| Genomes| Genetics*, 1(6):457–470, 2011.

[22] Benjamin W Domingue, Sam Trejo, Emma Armstrong-Carter, and Elliot M Tucker-Drob. Interactions between polygenic scores and environments: Methodological and conceptual challenges. *Sociological Science*, 7:465–486, 2020.

[23] Bjarni J Vilhjálmsson, Jian Yang, Hilary K Finucane, Alexander Gusev, Sara Lindström, Stephan Ripke, Giulio Genovese, Po-Ru Loh, Gaurav Bhatia, Ron Do, et al. Modeling linkage disequilibrium increases accuracy of polygenic risk scores. *The American Journal of Human Genetics*, 97(4):576–592, 2015.

[24] International HapMap 3 Consortium et al. Integrating common and rare genetic variation in diverse human populations. *Nature*, 467(7311):52, 2010.

[25] Lambert H Lumey, Aryeh D Stein, and Ezra Susser. Prenatal famine and adult health. *Annual Review of Public Health*, 32:237–262, 2011.

[26] Douglas Almond and Bhashkar Mazumder. Health capital and the prenatal environment: the effect of Ramadan observance during pregnancy. *American Economic Journal: Applied Economics*, 3(4):56–85, 2011.
